# Retraction Note: Online positive parenting programme for promoting parenting competencies and skills: randomised controlled trial

**DOI:** 10.1038/s41598-024-66589-7

**Published:** 2024-07-15

**Authors:** Sararat Tuntipuchitanon, Ing-on Kangwanthiti, Ketsupar Jirakran, Pon Trairatvorakul, Weerasak Chonchaiya

**Affiliations:** 1 Division of Growth and Development, Department of Paediatrics, Faculty of Medicine, Chulalongkorn University, Bangkok, Thailand; 2 Maximizing Thai Children’s Developmental Potential Research Unit, Division of Growth and Development, Department of Paediatrics, Sor Kor Building, 11th floor, King Chulalongkorn Memorial Hospital, Faculty of Medicine, Chulalongkorn University, 1873 Rama IV Road, Pathumwan, Bangkok, 10330 Thailand

Retraction of: *Scientific Reports* 10.1038/s41598-022-10193-0, published online 19 April 2022

The authors have retracted this article. The study reported used a translated version of the Parenting Stress Index-Short Form (PSI-SF). After publication of this article the authors were made aware that the copyright owner of the PSI-SF had not granted permission for this use.

All authors agree with this retraction.

